# Investigating the prognostic value of mammographic breast density and mammographic tumor appearance in women with invasive breast cancer: The Malmö Diet and cancer study

**DOI:** 10.1016/j.breast.2023.05.004

**Published:** 2023-05-23

**Authors:** Li Sturesdotter, Anna-Maria Larsson, Sophia Zackrisson, Hanna Sartor

**Affiliations:** a Department of Translational Medicine, Diagnostic Radiology, Lund University, Lund, Sweden; b Department of Medical Imaging and Physiology, Skåne University Hospital, Lund/Malmö, Sweden; c Department of Clinical Sciences Lund, Division of Oncology, Lund University, Lund, Sweden; d Unilabs Breast Unit, Skåne University Hospital, Malmö, Sweden

**Keywords:** Breast cancer, Mammography, Epidemiology, Breast density, Survival, Prognosis

## Abstract

**Background:**

High breast density is a risk factor for breast cancer. However, whether density is a prognostic factor is debatable. Also, tumor appearances are related to tumor characteristics. Here we investigate the relationship between breast cancer-specific survival and mammographic breast density and mammographic tumor appearances.

**Methods:**

Women in the Malmö Diet and Cancer study with invasive breast cancer 1991–2014 were included (n = 1116). Mammographic information, patient and tumor characteristics, vital status, and causes of death were collected through 2018. Breast cancer-specific survival was assessed with Kaplan-Meier estimates and Cox proportional hazard models. Analyses were adjusted for established prognostic factors and stratified by detection mode.

**Results:**

High breast density did not significantly impact breast cancer-specific survival. However, there may be increased risk in women with dense breasts and screening-detected tumors (HR 1.45, CI 0.87–2.43). Neither did tumor appearance impact breast cancer-specific survival at long-term follow-up.

**Conclusions:**

Breast cancer prognosis in women with high breast density on mammography does not seem impaired compared to women with less dense breasts, once the cancer is established. Neither does mammographic tumor appearance seem to inflict on prognosis, findings that can be of value in the management of breast cancer.

## Highlights

• Breast cancer survival studied in relation to breast density and tumor appearances.
• Large study within a prospective population-based cohort with long-term follow-up.
• No certain evidence of decreased survival for women with dense breasts.
• No certain evidence of decreased survival due to certain tumor appearances.

## 1 Background

High breast density is a strong risk factor for breast cancer, which has created great interest in this field [1,2]. Dense tissue appears white on a mammogram (radio-opaque), due to the abundance of glandular and connective tissue, and can make it difficult to detect cancer which is commonly also radio-opaque – a phenomenon called “the masking effect” [1]. Consequently, cancer in dense breasts could carry a worse prognosis as they may be discovered at a later stage of disease [3]. Cancers arising in dense breasts tend to be larger and accompanied by positive lymph nodes, compared to cancers in less dense breasts [4], which has also been shown by our research group [5]. The masking effect might partly explain these findings. However, there is also evidence of microenvironmental alterations in dense tissue that may promote carcinogenesis in a multifactorial manner [6]. The importance of density for prognosis is not fully clarified [4,7,8], but is of great importance to investigate, since it concerns numerous women worldwide, and might affect breast cancer management.

The mammographic appearance of breast tumors is also evaluated in clinical practice. Tumor appearance is associated with histopathological tumor characteristics [[9], [10], [11], [12], [13]]. E.g., spiculation is associated with favorable characteristics, which has been shown by our group [14] and others [11,12,15]. The spiculated tumors are often of molecular Luminal A-subtype, known for carrying the best prognosis [14,16]. The better survival rate among women with spiculated tumors [[16], [17], [18]], and worse for women with certain tumor-associated calcifications have been reported [18,19]. Based on the results from our previous study [14], we hypothesize that spiculated tumor appearance implies improved survival. Another prognostic factor to consider is the detection method – whether the tumor was discovered during a screening round or diagnosed clinically. It is well-known that clinically detected cancers carry a worse prognosis [20]. Hence, the mode of detection is important to account for. As far as we know, the combination of breast density, tumor appearance and mode of detection has not been thoroughly studied in relation to breast cancer survival.

In this study, we aim to investigate breast density and tumor appearance, and their relation to breast cancer survival, using the large prospective Malmö Diet and Cancer Study (MDCS) [21].

## 2 Material and methods

### 2.1 Study population

The MDCS [21] was approved by the Ethics committee at Lund University (Official Records No. LU 51–90) [22]. It included inhabitants in Malmö 1991–1996, of which 17,035 were women [23]. At baseline, participants were physically examined and completed a detailed questionnaire [21]. The MDCS database includes information on cancer diagnoses, vital status, and causes of death, and is updated regularly with information from the Swedish Cancer Register and the Swedish Cause of Death Register [24]. We have conducted a retrospective study with data from the MDCS. All women in the cohort diagnosed with breast cancer from 1991 until the end of 2014 were identified. Women with prevalent breast cancer at baseline (n = 572), bilateral breast cancer (n = 21), and non-invasive cancer (Carcinoma In Situ) (n = 105) were excluded. After the application of exclusion criteria, 1116 women remained eligible for inclusion.

Baseline information [including use of hormone replacement therapy (HRT) and body mass index (BMI)], and factors related to breast cancer diagnosis: mammographic information, pathology reports, and information from medical records, were collected. Follow-up data, including causes of death, was available until the December 31, 2018. The endpoint was breast cancer-specific survival. Vital status was defined as alive/dead from other causes or dead due to breast cancer. Dead due to breast cancer was appointed when breast cancer was either the underlying or contributing cause of death. The study was carried out in accordance with the declaration of Helsinki and was approved by the Ethics Review Board in Lund, Sweden (Official Records Nos. 652/2005, 166/2007 and 2014/830) and by the Swedish Ethical Review Authority (2022-04473-02). It includes a subset of the women in the MDCS cohort, their informed consent was obtained at the baseline examination.

### 2.2 Clinical data

**Fig. 1:**
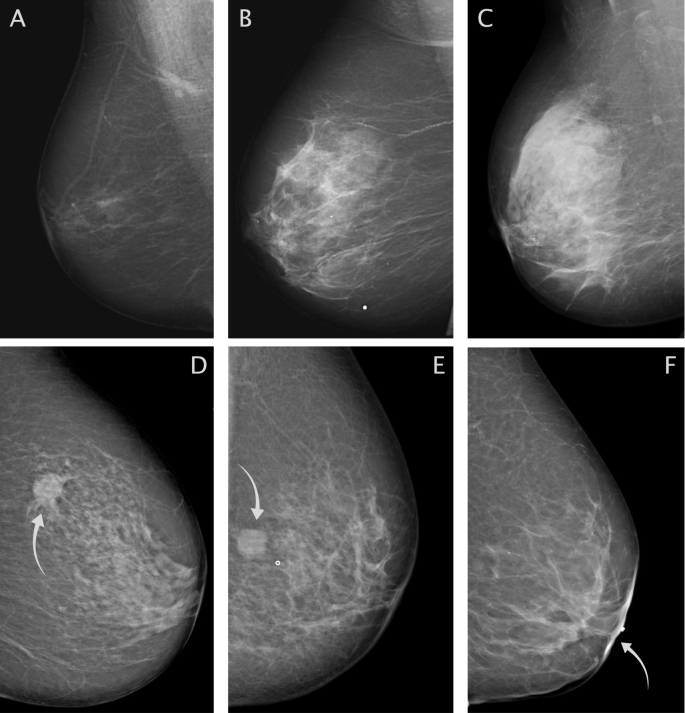
**Breast density and tumor appearances.** Upper row: Mammograms illustrating variations in breast density. From left to right: A) fat involuted, B) moderately dense, and C) dense breast parenchyma. Bottom row: Mammograms illustrating different tumor appearances. From left to right: D) spiculated mass, E) distinct mass, and F) ill-defined mass (located retro-mamillary with retracted nipple).

At the Department of Breast Radiology in Malmö, screening mammography is double-read by two breast radiologists, while re-call examinations in association with screening are single-read. Clinically detected cases are routinely single-read by one breast radiologist. For women in this study, the cancers were detected at screening or clinically. The clinically detected cancers include cancers diagnosed between screening rounds, so-called “interval cancers”. Breast density and tumor appearance were retrospectively collected from the mammography report at the time of diagnosis, by SZ (breast radiologist) and/or HS (at the time a radiology resident with special training in breast radiology). If the original report was incomplete regarding density and/or tumor appearance, the original mammogram was reviewed retrospectively by SZ and/or HS. If the report was inconclusive and the mammogram couldn't be located, the case was classified as missing [24]. A total of 69 (6.2% of the population) and 107 cases (9.6% of the population) were classified as missing for breast density and tumour appearance, respectively. The predominant reason was that the mammograms were old, analogous and could not be found in the archives. Hence, the missing cases are expected to be randomly distributed. In the clinic, density is routinely classified qualitatively into three categories: fat-involuted, moderately dense, and dense breast parenchyma (Fig. 1, upper row). These categories correspond to The Breast Imaging Reporting and Data System (BI-RADS) [25] 4th edition: “fat involuted” to BI-RADS 1 (almost entirely fatty), “moderately dense” to BI-RADS 2 and 3 (scattered fibroglandular density and heterogeneously dense), and “dense” to BI-RADS 4 (extremely dense). For a subset of the population (n = 376) diagnosed 2008–2014, an additional density assessment according to BI-RADS 5th edition (a = almost entirely fatty, b = scattered areas of fibroglandular density, c = heterogeneously dense, and d = extremely dense) was made retrospectively by HS.

The tumor appearances were divided into five groups, according to an adjusted model by Luck et al. [26], previously applied by our group [24]: distinct mass, ill-defined mass, spiculation, microcalcification, and tissue abnormality. Only the most dominant tumor appearance was considered. Examples of spiculation, distinct mass and ill-defined mass are illustrated in Fig. 1, bottom row. The tissue abnormality group includes the rather infrequent appearances asymmetric density, and architectural distortion. Tumor characteristics were collected from medical records or tissue microarrays (TMA) constructed for the MDCS cohort [27]. Tumor size and axillary lymph node involvement (ALNI) were collected from medical records. Histological grade [28] was re-assessed for tumors from the first part of the study (from 1991 to the end of 2004) and thereafter collected from medical records. Status for estrogen receptor (ER) and human epidermal growth factor 2 (HER2) was assessed on TMA 1991–2007 and extracted from medical records from 2008 and onwards [27].

### 2.3 Statistical analyses

Descriptive statistics were used to visualize the study population. Kaplan-Meier estimate and Cox's proportional hazard regression were used to study the impact of breast density and tumor appearance on breast cancer-specific survival. The proportional hazards assumption was confirmed using a log-minus-log plot. Cox regression analyses yielded hazard ratios (HR) and 95% confidence intervals (CI). Cox analyses regarding breast density were made with the least dense categories combined (fat involuted + moderately dense parenchyma) compared to the densest category (dense breast parenchyma). Cox analyses were also made separately for the women (n = 376) with density assessment according to BIRADS 5th edition. In these analyses, categories a & b and c & d (as described above) were combined. Survival analyses were made at the longest possible follow-up for each woman, and at 5 years after diagnosis – a commonly used time point for comparison of cancer survival [29,30]. The 5-year analyses are attached as appendices. All Cox analyses were first computed for the whole population and then stratified by mode of detection (screening or clinically detected). Cox analyses were adjusted for age at diagnosis, tumor size, ALNI, histological grade, and ER status – all established prognostic factors in breast cancer. Breast density analyses were in addition adjusted for BMI and HRT at baseline, which can affect breast density [31]. Cox analyses regarding tumor appearance were not adjusted for BMI and HRT but for breast density, which can influence the perception of mammographic tumor appearance [32].

For adjustments, age at diagnosis was used as a continuous variable, tumor size was dichotomized (≤20 mm or > 20 mm), ALNI was binarized (present or not), histological grade was divided into three ordinal categories, ER was binarized (positive or negative), BMI was used a continuous variable, HRT was binarized, and breast density was divided into three ordinal categories based on clinical practice. A p-value of less than 0.05 was considered statistically significant. All calculations were made with Stata, version 16.1.

## 3 Results

Table 1 shows patient and tumor characteristics in relation to breast cancer survival. The median follow-up time was 10.7 years (range 0–27.1). A total of 202 women had died from breast cancer, their median follow-up time was 5.3 years. For the remaining 914 women, the median follow-up time was 11.7 years. Of these, 214 had died from other causes, and four had emigrated. Spiculated tumors were (in terms of percentages) more common in women who were alive/dead from other causes, while ill-defined tumors were more common in women who had died from breast cancer. A greater proportion of the women who had died from breast cancer were diagnosed clinically, had larger tumors, higher histological grade, and ALNI (Table 1).

**Table 1:** Patient and tumor characteristics in relation to breast cancer survival.

	Alive or dead from other causes (n = 914)	Dead due to breast cancer (n = 202)
**Age at diagnosis, median (range)**	66 (46–91)	66 (45–91)
**Follow-up time in years, median (range)**	11.7 (0–27.1)	5.3 (0–21.9)
**Breast density**		
Fat involuted	157 (18.0)	28 (15.9)
Moderately dense	424 (48.7)	81 (46.0)
Dense	290 (33.3)	67 (38.1)
Missing	43	26
**BIRADS 5th edition (available for a subset of the study population, n = 376)**		
Almost entirely fatty	77 (23.2)	11 (25.0)
Scattered areas of fibroglandular density	121 (36.4)	16 (36.4)
Heterogeneously dense	107 (32.2)	14 (31.8)
Extremely dense	27 (8.1)	3 (6.8)
Missing	582	158
**Mammographic appearance**		
Distinct mass	228 (27.2)	38 (22.4)
Ill-defined mass	155 (18.5)	48 (28.2)
Spiculated	358 (42.7)	58 (34.1)
Calcifications	70 (8.3)	13 (7.6)
Tissue abnormality	28 (3.3)	13 (7.6)
Missing	75	32
**Mode of detection**		
Screening detection	492 (54.4)	63 (31.7)
Clinical detection	413 (45.6)	136 (68.3)
Missing	9	3
**Tumor size**		
≤20 mm	669 (76.4)	77 (43.3)
>20 mm	207 (23.6)	101 (56.7)
Missing	38	24
**Axillary lymph node involvement (ALNI)**		
No	608 (73.9)	70 (39.8)
Yes	215 (26.1)	106 (60.2)
Missing	91	26
**Histological grade**		
I	249 (29.4)	20 (11.7)
II	411 (48.6)	70 (40.9)
III	186 (22.0)	81 (47.4)
Missing	68	31
**Estrogen receptor**		
Negative	83 (10.2)	25 (16.4)
Positive	728 (89.8)	127 (83.6)
Missing	103	50
**Hormone replacement therapy at baseline**		
No	666 (73.1)	157 (77.7)
Yes	245 (26.9)	45 (22.3)
Missing	3	0
**Body mass index at baseline, median (range)**	24.8 (16.2–42.1)	25.4 (17.1–46.1)
**HER2 status**		
Negative	692 (91.9)	120 (84.5)
Positive	61 (8.1)	22 (15.5)
Missing	161	60

Data presented as count (percent) unless otherwise stated.

### 3.1 Breast density

**Fig. 2:**
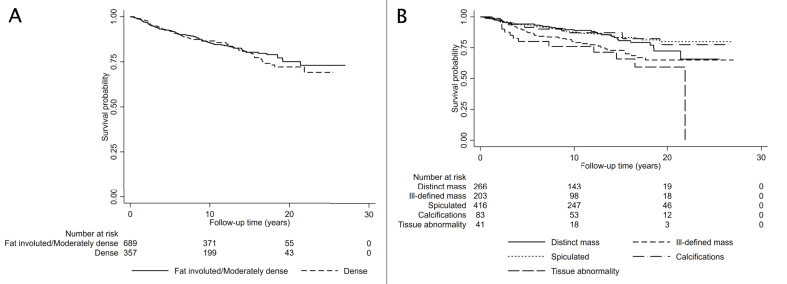
**Breast density and tumor appearance in relation to survival** A) Survival status in relation to breast density illustrated in a Kaplan-Meier graph. B) Survival status in relation to tumor appearance illustrated in a Kaplan-Meier graph.

**Fig. A.1:**
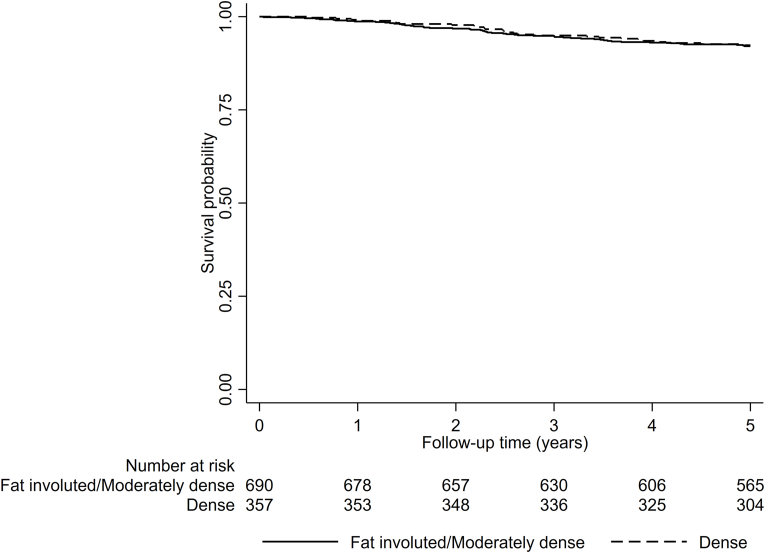
**Breast density in relation to 5-year survival.** Survival status 5 years after diagnosis in relation to breast density illustrated in a Kaplan-Meier graph.

In Fig. 2, panel A, a Kaplan-Meier graph visualizes vital status over time for the entire study population, in relation to breast density. No major differences were seen between the two density groups: fat involuted + moderately dense and dense breast parenchyma. In the subsequent Cox analysis (Table 2), we found no significant differences between the density groups, neither for the entire population nor after stratification by detection mode. For the entire population, the HR for breast cancer death in women with dense breast parenchyma was 1.08 (CI 0.80–1.47) (Table 2). The HR was virtually unchanged after adjustment for age at diagnosis, BMI, HRT, tumor size, ALNI, histological grade, and ER+, HR _adj_ 1.15 (CI 0.79–1.68) (Table 2). In stratified analysis, for women with screening-detected tumors and dense breasts the HR was 1.45 (CI 0.87–2.43) (Table 2). Moreover, survival analysis for the women assessed with BI-RADS 5th edition did not show significant associations (Table 3). The HR and HR_adj_ of breast cancer-specific death for the two denser categories combined (heterogeneously dense and extremely dense) were 0.93 (CI 0.51–1.71) and 0.80 (CI 0.37–1.74), respectively, compared to the least dense categories (almost entirely fatty and scattered areas of fibroglandular density) (Table 3). Analyses stratified by detection mode likewise yielded insignificant results (Table 3). Additional survival analyses at 5 years after diagnosis were calculated for the whole population and the subset with BI-RADS 5th edition assessment; see Figure A1 and Table A.1 & A.2 in the appendices. At this shorter follow-up, no significant differences in breast cancer-specific survival were identified.

**Table 2:** Breast density in relation to survival.

Entire study population	Alive^b^	Dead^c^				
Breast density	n (%)		HR (95% CI)	p-value 0.609	HR_adj_^a^(95% CI)	p-value 0.466
Fat-involuted/Moderately dense	581 (66.7)	109 (61.9)	1.0 (Ref.)		1.0 (Ref.)	
Dense	290 (33.3)	67 (38.1)	1.08 (0.80–1.47)		1.15 (0.79–1.68)	
Observations			1046		864	
**Screening-detected**	Alive^b^	Dead^c^				
Breast density	n (%)		HR (95% CI)	p-value 0.153	HR_adj_^a^(95% CI)	p-value 0.465
Fat-involuted/Moderately dense	329 (69.4)	34 (56.7)	1.0 (Ref.)		1.0 (Ref.)	
Dense	145 (30.6)	26 (43.3)	1.45 (0.87–2.43)		1.29 (0.65–2.53)	
Observations			534		439	
**Clinically detected**	Alive^b^	Dead^c^				
Breast density	n (%)		HR (95% CI)	p-value 0.359	HR_adj_^a^(95% CI)	p-value 0.872
Fat-involuted/Moderately dense	251 (63.4)	75 (64.7)	1.0 (Ref.)		1.0 (Ref.)	
Dense	145 (36.6)	41 (35.3)	0.84 (0.57–1.23)		1.04 (0.65–1.65)	
Observations			512		424	

a Adjusted for age at diagnosis, BMI, HRT, tumor size, ALNI, histological grade and ER+.

b Alive or dead from other causes.

c Dead due to breast cancer.

**Table 3:** Breast density according to BIRADS 5th edition in relation to survival, in a subset of the study population (n = 376).

Entire study population	Alive^b^	Dead^c^				
Breast density	n (%)		HR (95% CI)	p-value 0.825	HR_adj_^a^(95% CI)	p-value 0.582
Almost entirely fatty/scattered areas of fibroglandular density	198 (59.6)	27 (61.4)	1.0 (Ref.)		1.0 (Ref.)	
Heterogeneously dense/extremely dense	134 (40.4)	17 (38.6)	0.93 (0.51–1.71)		0.80 (0.37–1.74)	
Observations			376		332	
**Screening detected**	Alive^b^	Dead^c^				
Breast density	n (%)		HR (95% CI)	p-value 0.894	HR_adj_^a^(95% CI)	p-value 0.681
Almost entirely fatty/scattered areas of fibroglandular density	105 (66.9)	6 (66.7)	1.0 (Ref.)		1.0 (Ref.)	
Heterogeneously dense/extremely dense	52 (33.1)	3 (33.3)	1.10 (0.27–4.41)		1.50 (0.79–10.47)	
Observations			166		156	
**Clinically detected**	Alive^b^	Dead^c^				
Breast density	n (%)		HR (95% CI)	p-value 0.323	HR_adj_^a^(95% CI)	p-value 0.440
Almost entirely fatty/scattered areas of fibroglandular density	93 (53.1)	21 (60.0)	1.0 (Ref.)		1.0 (Ref.)	
Heterogeneously dense/extremely dense	82 (46.9)	14 (40.0)	0.71 (0.36–1.40)		0.71 (0.29–1.70)	
Observations			210		176	

a Adjusted for age at diagnosis, BMI, HRT, tumor size, ALNI, histological grade and ER+.

b Alive or dead from other cause.

c Dead due to breast cancer.

### 3.2 Tumor appearance

**Fig. A.2:**
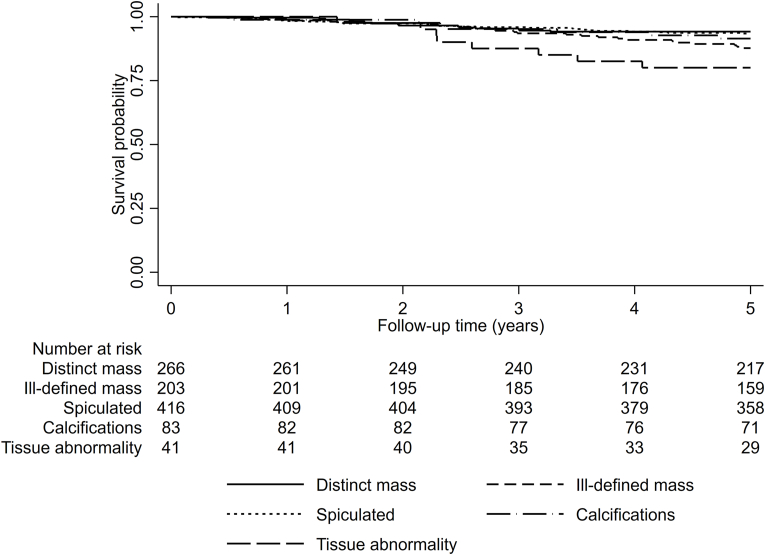
**Tumor appearance in relation to 5-year survival.** Survival status 5 years after diagnosis in relation to tumor appearance illustrated in a Kaplan-Meier graph.

Fig. 2, panel B, shows a Kaplan-Meier survival graph for the entire study population in relation to tumor appearances. Cox analysis for long-term follow-up (Table 4) showed increased HR for breast cancer death in ill-defined mass and tissue abnormality [1.64 (CI 1.07–2.51), and 2.37 (CI 1.26–4.46), respectively] in crude analysis. However, after adjustments, results were insignificant [HR _adj_ 1.48 (CI 0.88–2.49) and 2.02 (CI 0.89–4.60), respectively] (Table 4). In the stratified analysis on screening-detected cases, no significant findings were found. For the clinically detected cancers only, ill-defined mass and tissue abnormality had a worse prognosis in crude analysis, but again this result was not significant in adjusted analysis (Table 4). The spiculated tumor appearance did not have a significant impact on breast cancer-specific survival – neither in the whole group [HR_adj_ 1.24 (CI 0.75–2.03)] nor after stratification on detection mode, when studied at long-term follow-up (Table 4). Tumor appearances and breast cancer survival were also investigated at 5 years after diagnosis; see Figure A2 and Table A3 in the appendices. Worse prognosis for ill-defined mass and tissue abnormality was again found in crude, but not in adjusted analyses (Table A3). At 5 years after diagnosis, results were insignificant concerning spiculated tumors for the entire group and among women with screening-detected cancer (Table A3). However, for women with clinically detected tumors, spiculated appearance was significantly associated with increased risk of breast cancer death compared to distinct mass, with an HR_adj_ of 2.77 (1.03–7.46), Table A3.

**Table 4:** Mammographic tumor appearance in relation to survival.

Entire study population	Alive^b^	Dead^c^				
Mammographic appearance	n (%)		HR (95% CI)	p-value 0.001	HR_adj_^a^(95% CI)	p-value 0.438
Distinct mass	228 (27.2)	38 (22.4)	1.0 (Ref.)		1.0 (Ref.)	
Ill-defined mass	155 (18.5)	48 (28.2)	1.64 (1.07–2.51)		1.48 (0.88–2.49)	
Spiculated	358 (42.7)	58 (34.1)	0.90 (0.60–1.35)		1.24 (0.75–2.03)	
Calcifications	70 (8.3)	13 (7.6)	0.91 (0.48–1.70)		1.36 (0.61–3.03)	
Tissue abnormality	28 (3.3)	13 (7.6)	2.37 (1.26–4.46)		2.02 (0.89–4.60)	
Observations			1009		818	
**Screening detected**	Alive^b^	Dead^c^				
Mammographic appearance	n (%)		HR (95% CI)	p-value 0.483	HR_adj_^a^(95% CI)	p-value 0.915
Distinct mass	101 (21.1)	13 (23.6)	1.0 (Ref.)		1.0 (Ref.)	
Ill-defined mass	79 (16.5)	9 (16.4)	0.85 (0.36–1.99)		0.89 (0.31–2.53)	
Spiculated	230 (48.1)	23 (41.8)	0.76 (0.39–1.50)		0.89 (0.40–1.97)	
Calcifications	56 (11.7)	6 (10.9)	0.74 (0.28–1.94)		0.87 (0.25–3.01)	
Tissue abnormality	12 (2.5)	4 (7.3)	1.96 (0.64–6.03)		1.84 (0.38–8.89)	
Observations			533		430	
**Clinically detected**	Alive^b^	Dead^c^				
Mammographic appearance	n (%)		HR (95% CI)	p-value 0.021	HR_adj_^a^(95% CI)	p-value 0.420
Distinct mass	126 (35.0)	25 (21.7)	1.0 (Ref.)		1.0 (Ref.)	
Ill-defined mass	76 (21.1)	39 (33.9)	2.06 (1.25–3.40)		1.77 (0.94–3.33)	
Spiculated	128 (35.6)	35 (30.4)	1.24 (0.74–2.08)		1.57 (0.83–2.99)	
Calcifications	14 (3.9)	7 (6.1)	1.75 (0.76–4.06)		1.77 (0.58–5.40)	
Tissue abnormality	16 (4.4)	9 (7.8)	2.45 (1.14–5.26)		2.18 (0.79–6.02)	
Observations			475		387	

a Adjusted for age at diagnosis, breast density, tumor size, ALNI, histological grade and ER+.

b Alive or dead from other cause.

c Dead due to breast cancer.

How adjustment factors relate to survival, 5-year survival, spiculated tumor appearance and dense breast parenchyma is presented in Table A4 in the appendices.

## 4 Discussion

In this large study including 1116 women with invasive breast cancer, we found no evidence of a significant association between mammographic breast density and tumor appearance, and breast cancer-specific survival. However, regarding screening-detected tumors the survival was impaired for the women with the densest breasts. Although not significant, this might be suggestive of increased mortality in this group, but the results should be interpreted with caution. Several other studies have neither identified impaired survival in women with dense breasts [3,20,[33], [34], [35], [36], [37]]. On the contrary, some studies have reported such an association [8,38,39]. Breast density and survival have previously been studied by Olsson et al. [38] within the same cohort as the present study. However, we included an additional 497 women and have data during a longer follow-up period. Olsson et al. reported impaired survival in women with dense breasts compared to women with fatty breasts. A key difference is that we combined women with fatty and moderately dense breasts and compared them to those with the densest breasts. We believe this is clinically more relevant, as the densest breast parenchyma carries the most increased breast cancer risk. In addition, we performed density analyses based on BI-RADS 5th edition, which enables a comparison with the internationally used standard. Neither in these analyses did we find evidence for impaired survival in women with denser breasts, when taking the whole study population into account. Beyond differences in methodology, sample size, and follow-up, the aspect of time passing is important to consider. Better treatment options have emerged during the follow-up period, such as aromatase inhibitors and monoclonal antibodies targeted at the HER2 receptor, in combination with new chemotherapy regimens. This has likely improved survival for many women in the study. If high breast density is prognostically unfavorable after all, its effect might have been diminished over time due to the implementation of new and improved treatments.

Nevertheless, a large review [4] concluded that most studies are in favor of a non-association between high density and survival. With our present study, we mainly add support to this standpoint. Our study is an important addition to the field due to its large size and long follow-up. However, studies with even longer follow up are needed to confirm the results, since for example, ER + tumors are known for their ability to metastasize and cause mortality decades after the first diagnosis [40,41]. Beyond academia, high breast density is of great interest in society. Although still a strong risk factor for breast cancer development, once a cancer is diagnosed in women with dense breasts, these women do not seem to have a worse prognosis. Distinguish between risk and prognosis can be tricky, and healthcare professionals must convey this knowledge in an understandable manner.

Previous studies have reported a better survival rate for spiculated tumors [[16], [17], [18]]. However, adjustment factors and other methodological issues have varied in these studies, and none of them have adjusted for breast density. This is potentially very important as it might influence the perception of breast lesions [32,42]. Surprisingly, we detected a somewhat worse prognosis at 5-year follow-up for women with clinically detected spiculated tumors, compared to distinct mass, after adjusting for several confounding factors. However, this finding should be interpreted with great caution as there was no significant association in the crude analysis. The sample size was also rather small, which adds to further uncertainty. If such an association exists, clinically detected spiculated tumors might differ from the screening-detected ones somehow. Larger studies are needed to investigate this matter. Impaired survival in women with tumors presenting with calcifications on mammography [43], and particularly for those with calcifications with a casting-type constitution have been reported [18,19,44]. Again, none of these studies adjusted for breast density. We could not affirm these findings. Microcalcifications were rarely described in detail in the present study, making it impossible to distinguish different types of calcifications. At long-term follow-up, results were insignificant regarding all tumor appearances. The reasons for this might be numerous. Breast cancer is a multifactorial disease, and the influence of tumor appearances might not be strong enough to affect long-term prognosis, even though they are associated with cancer characteristics at diagnosis. The way of categorizing appearances may have inflicted on the possibility to distinguish differences between some of the subgroups. Further, the subgroups were partly small, which can affect statistical power and contribute to the uncertainty of results. Moreover, assessing tumor appearance solely on mammography has its limitations. Future investigations on other imaging modalities could potentially prove different. Imaging is central in breast cancer care, benefits over pathology from being non-invasive and is introduced in the very beginning of the diagnostic chain. Even if histopathology adds crucial diagnostic information, we believe that the images withhold important information that may improve prognostic and predictive clinical decision making at an earlier stage.

A strength of this study is that it is based on a large population-based cohort. It includes more than a thousand women with invasive breast cancer for whom extensive information is available. Furthermore, the follow-up time is long, from the inclusion period 1991–1996 until the end of 2018, and high coverage in Swedish national registries guarantees high-quality data. However, there are also a few weaknesses. Qualitative assessment of mammograms might introduce variation between radiologists. But at the same time, this reflects everyday clinical practice. For temporal aspects of breast density, it would have been interesting to have studied density on mammograms acquired before breast cancer diagnosis, however this was not possible in the MDCS cohort. The study only included women from a single institution, which limits the representativeness. Women in the MDCS are generally healthier and have a slightly higher educational level than the average female population, which can also affect representativeness. Despite this, the distribution within breast cancer subtypes, density and tumor appearances resembles that in everyday practice [14]. Also, the internal comparisons should not be affected.

## 5 Conclusion

Breast density, assessed at the time of cancer diagnosis, does not influence breast cancer-specific survival for women within the MDCS cohort with certainty. Also, by using a clinically oriented way of categorizing tumor appearances, we found no impact of mammographic tumor appearance on long-term breast cancer survival. For health care professionals, one implication could be that women with dense breasts or certain tumor features do not require additional surveillance regarding prognosis once breast cancer is diagnosed. However, this might have to be re-evaluated as other imaging modalities are being increasingly used.

## Data statement

The dataset from the Malmö Cohorts supporting the conclusions of this article was used under a license and is not available as an open source. Please visit their website for more information [45].

## Ethics approval

This study was carried out in accordance with the declaration of Helsinki. It was approved by the Ethics Review Board in Lund, Sweden (Official Records Nos. 652/2005, 166/2007 and 2014/830) and by the Swedish Ethical Review Authority (2022-04473-02). It includes a subset of the women in the MDCS cohort, and their informed consent was obtained at baseline examination.

## Funding

This work was supported by The South Swedish Health Care Region and Region Skåne. The funding sources were not involved in the execution of the work.

**Table A.1:** Breast density in relation to 5-year survival.

Entire study population	Alive^b^	Dead^c^				
Breast density	n (%)		HR (95% CI)	p-value 0.862	HR_adj_^a^(95% CI)	p-value 0.924
Fat-involuted/Moderately dense	637 (65.9)	53 (66.3)	1.0 (Ref.)		1.0 (Ref.)	
Dense	330 (34.1)	27 (33.8)	0.96 (0.60–1.52)		1.03 (0.56–1.90)	
Observations			1047		864	
**Screening detected**	Alive^b^	Dead^c^				
Breast density	n (%)		HR (95% CI)	p-value 0.431	HR_adj_^a^(95% CI)	p-value 0.439
Fat-involuted/Moderately dense	351 (68.3)	12 (60.0)	1.0 (Ref.)		1.0 (Ref.)	
Dense	163 (31.7)	8 (40.0)	1.43 (0.59–3.50)		1.66 (0.46–6.05)	
Observations			534		439	
**Clinically detected**	Alive^b^	Dead^c^				
Breast density	n (%)		HR (95% CI)	p-value 0.319	HR_adj_^a^(95% CI)	p-value 0.699
Fat-involuted/Moderately dense	285 (63.1)	41 (68.3)	1.0 (Ref.)		1.0 (Ref.)	
Dense	167 (36.9)	19 (31.7)	0.76 (0.44–1.30)		0.87 (0.43–1.77)	
Observations			512		424	

a Adjusted for age at diagnosis, BMI, HRT, tumor size, ALNI, histological grade and ER+.

b Alive or dead from other causes.

c Dead due to breast cancer.

**Table A.2:** Breast density according to BIRADS 5th edition in relation to 5-year survival, in a subset of the study population (n = 376).

Entire study population	Alive^b^	Dead^c^				
Breast density	n (%)		HR (95% CI)	p-value 0.834	HR_adj_^a^(95% CI)	p-value 0.733
Almost entirely fatty/scattered areas of fibroglandular density	206 (59.7)	19 (61.3)	1.0 (Ref.)		1.0 (Ref.)	
Heterogeneously dense/extremely dense	139 (40.3)	12 (38.7)	0.93 (0.45–1.91)		0.84 (0.32–2.24)	
Observations			376		332	
**Screening detected**	Alive^b^	Dead^c^				
Breast density	n (%)		HR (95% CI)	p-value 0.379	HR_adj_^a^(95% CI)	p-value 0.178
Almost entirely fatty/scattered areas of fibroglandular density	108 (67.5)	3 (50.0)	1.0 (Ref.)		1.0 (Ref.)	
Heterogeneously dense/extremely dense	52 (32.5)	3 (50.0)	2.05 (0.41–10.16)		8.05 (0.39–167.58)	
Observations			166		156	
**Clinically detected**	Alive^b^	Dead^c^				
Breast density	n (%)		HR (95% CI)	p-value 0.270	HR_adj_^a^(95% CI)	p-value 0.334
Almost entirely fatty/scattered areas of fibroglandular density	98 (53.0)	16 (64.0)	1.0 (Ref.)		1.0 (Ref.)	
Heterogeneously dense/extremely dense	87 (47.0)	9 (36.0)	0.63 (0.28–1.43)		0.57 (0.19–1.77)	
Observations			210		176	

a Adjusted for age at diagnosis, BMI, HRT, tumor size, ALNI, histological grade and ER+.

b Alive or dead from other causes.

c Dead due to breast cancer.

**Table A.3:** Mammographic tumor appearance in relation to 5-year survival.

Entire study population	Alive^b^	Dead^c^				
Mammographic appearance	n (%)		HR (95% CI)	p-value 0.009	HR_adj_^a^(95% CI)	p-value 0.358
Distinct mass	251 (27.0)	15 (18.5)	1.0 (Ref.)		1.0 (Ref.)	
Ill-defined mass	179 (19.3)	24 (29.6)	2.07 (1.09–3.95)		1.67 (0.70–3.98)	
Spiculated	389 (41.9)	27 (33.3)	1.11 (0.59–2.09)		1.87 (0.80–4.35)	
Calcifications	76 (8.2)	7 (8.6)	1.45 (0.59–3.55)		2.45 (0.74–8.10)	
Tissue abnormality	33 (3.6)	8 (9.9)	3.58 (1.52–8.45)		3.13 (0.90–10.84)	
Observations			1009		818	
**Screening detected**	Alive^b^	Dead^c^				
Mammographic appearance	n (%)		HR (95% CI)	p-value 0.351	HR_adj_^a^(95% CI)	p-value 0.709
Distinct mass	111 (21.6)	3 (15.8)	1.0 (Ref.)		1.0 (Ref.)	
Ill-defined mass	84 (16.3)	4 (21.1)	1.72 (0.39–7.69)		2.38 (0.36–15.67)	
Spiculated	246 (47.9)	7 (36.8)	1.04 (0.27–4.02)		1.01 (0.16–6.43)	
Calcifications	59 (11.5)	3 (15.8)	1.84 (0.37–9.10)		2.57 (0.32–20.54)	
Tissue abnormality	14 (2.7)	2 (10.5)	4.89 (0.82–29.28)		3.50 (0.29–42.71)	
Observations			533		430	
**Clinically detected**	Alive^b^	Dead^c^				
Mammographic appearance	n (%)		HR (95% CI)	p-value 0.115	HR_adj_^a^(95% CI)	p-value 0.284
Distinct mass	139 (33.7)	12 (19.4)	1.0 (Ref.)		1.0 (Ref.)	
Ill-defined mass	95 (23.0)	20 (32.3)	2.15 (1.05–4.40)		1.87 (0.68–5.10)	
Spiculated	143 (34.6)	20 (32.3)	1.49 (0.73–3.06)		2.77 (1.03–7.46)	
Calcifications	17 (4.1)	4 (6.5)	2.40 (0.77–7.45)		2.38 (0.49–11.69)	
Tissue abnormality	19 (4.6)	6 (9.7)	3.10 (1.16–8.25)		3.72 (0.85–16.23)	
Observations			475		387	

a Adjusted for age at diagnosis, density, tumor size, ALNI, histological grade and ER+.

b Alive or dead from other causes.

c Dead due to breast cancer.

**Table A.4:** Relating prognostic factors and predictors to survival, 5-year survival, spiculated mammographic tumor appearance, and the densest breast density categories. Evaluating each of the variables one at a time.

	Survival		5-year survival		Spiculated		Dense breast parenchyma		BI-RADS 5 c + d	
	HR (95% CI)	p	HR (95% CI)	p	OR (95% CI)	p	OR (95% CI)	p	OR (95% CI)	p
Age at diagnosis	1.05 (1.03–1.06)	<0.001	1.06 (1.03–1.08)	<0.001	1.00 (0.99–1.00)	0.660	0.99 (0.99–0.99)	<0.001	1.01 (1.01–1.01)	<0.001
BMI	1.04 (1.01–1.07)	0.019	1.03 (0.99–1.08)	0.180	1.01 (1.00–1.01)	0.026	0.97 (0.97–0.98)	<0.001	0.99 (0.99–0.99)	0.009
Hormone replacement therapy	0.75 (0.54–1.04)	0.088	0.87 (0.55–1.40)	0.573	1.03 (0.97–1.10)	0.387	1.09 (1.03–1.16)	0.006	1.00 (0.96–1.05)	0.855
ER	0.58 (0.38–0.89)	0.012	0.31 (0.18–0.56)	<0.001	1.33 (1.21–1.47)	<0.001	1.00 (0.91–1.1)	0.982	1.01 (0.95–1.09)	0.714
Histological grade		<0.001		<0.001		<0.001		0.097		0.712
I	1.0 (Ref.)		1.0 (Ref.)		1.0 (Ref.)		1.0 (Ref.)		1.0 (Ref.)	
II	2.12 (1.29–3.48)		3.23 (1.12–9.35)		0.97 (0.90–1.04)		0.99 (0.93–1.07)		0.99 (0.94–1.04)	
III	5.36 (3.28–8.74)		12.70 (4.57–35.30)		0.81 (0.74–0.87)		0.92 (0.86–1.00)		1.01 (0.96–1.07)	
Tumor size >20 mm	3.96 (.2.95–5.35)	<0.001	4.27 (2.69–6.79)	<0.001	0.92 (0.86–0.98)	0.010	1.06 (1.00–1.13)	0.074	1.05 (1.01–1.10)	0.019
Axillary lymph node involvement (ALNI)	3.79 (2.80–5.12)	<0.001	5.31 (3.25–8.66)	<0.001	1.01 (0.95–1.08)	0.747	1.03 (0.971–1.1)	0.298	0.99 (0.94–1.03)	0.544

## Appendices

Please see separately attached files (Figure A.1 and A.2, and Table A.1 – A.4).
